# Soluble Flt-1 Gene Delivery in Acute Myeloid Leukemic Cells Mediating a Nonviral Gene Carrier

**DOI:** 10.1155/2013/752603

**Published:** 2013-01-10

**Authors:** Razieh Amini, Farid Azizi Jalilian, Abhi Veerakumarasivam, Syahril Abdullah, Ahmed S. Abdulamir, Fatemeh Nadali, Rozita Rosli

**Affiliations:** ^1^Medical Genetics Laboratory, Faculty of Medicine and Health Sciences, Universiti Putra Malaysia, 43400 Serdang, Selangor, Malaysia; ^2^Department of Medical Microbiology, Faculty of Medicine, Ilam University of Medical Sciences, Ilam 69316, Iran; ^3^UPM-MAKNA Cancer Research Laboratory, Institute of Bioscience, Universiti Putra Malaysia, 43400 Serdang, Selangor, Malaysia; ^4^Perdana University Graduate School of Medicine, Perdana University, 43400 Serdang, Selangor, Malaysia; ^5^Department of Microbiology, College of Medicine, Al-Nahrain University, Baghdad, Iraq; ^6^Hematology Department, School of Allied Health Medicine, Tehran University of Medical Sciences, Tehran, Iran

## Abstract

Vascular endothelial growth factor (VEGF) is a potent angiogenic factor involved in angiogenesis-mediated progression of acute myeloid leukemia (AML). Studies have reported the role of soluble form of fms-like tyrosine kinase (sFlT-1) delivery as an antitumor agent by inhibiting VEGF. This study investigates the outcome of delivery of a VEGF165 antagonist, soluble vascular endothelial growth factor receptor, namely sFLT-1, mediating lipofectamine 2000 in acute myeloid leukemic cells. A recombinant plasmid expressing sFLT-1 was constructed and transfected into the K562 and HL60 cells using lipofectamine 2000 transfection reagent. sFLT-1 expression/secretion in pVAX-sFLT-1 transfected cells was verified by RT-PCR and western blot. MTS assay was carried out to evaluate the effect of sFLT-1 on human umbilical vein endothelial cells and K562 and HL60 cells *in vitro*. Treatment with pVAX-sFLT-1 showed no association between sFLT-1 and proliferation of infected K562 and HL60 cells, while it demonstrated a significant inhibitory impact on the proliferation of HUVECs. The results of the current study imply that the combination of nonviral gene carrier and sFLT-1 possesses the potential to provide efficient tool for the antiangiogenic gene therapy of AML.

## 1. Introduction

 Acute myeloid leukemia (AML) is a clonal disorder associated with acquired genetic defects that occur in early hematopoietic precursor cells. AML is categorized by rapid growth of clonal abnormal myeloid cells which fail to differentiate into functional granulocyte or monocyte and accumulate in the blood, bone marrow, or other organs [1, 2]. Angiogenesis is a complex series of interdependent procedures, controlled by many different factors. The angiogenic process results from a shift in the balance of positive and negative mediators, cytokines, and growth factors [3–5].

Angiogenesis has been postulated to play a role in pathogenesis of AML. Research in pathogenesis of AML has identified vascular endothelial growth factor (VEGF) to be among the most specific and essential regulators of the process of angiogenesis [6, 7]. VEGF regulates angiogenesis by modulating processes such as endothelial proliferation, permeability, and survival. Recent studies show that acute myeloid cells express a rich presence of VEGF as well as its receptors which are implicated in facilitation progression and survival of acute myeloid leukemic progenitors [8, 9]. Studies show that VEGF-mediated autocrine and paracrine signals are involved in infiltration of leukemic cells in bone marrow, liver, and spleen as well as increased microvascular density in bone marrow of AML patients [10–12]. In addition, evidence brought forth shows a linear correlation of the expression of VEGF and its receptors by AML blasts with reported low rates remission and overall survival among AML patients [12, 13].

The application of gene therapy in conjunction with other treatment modalities provides a promising novel approach in management of numerous hematological malignancies as it provides a means of circumventing setbacks suffered by current treatment modalities. The dual approach may result in improving the response by patients to already available conventional treatment [14–16]. 

 Endogenous inhibitors of VEGF hold a lot of promise on approach in regulation of angiogenesis, a major factor implicated in growth, development, and survival of many hematological malignancies including AML. Soluble form of fms-like tyrosine kinase (sFLT-1), are produced endogenously by alternative splicing of the FLT-1 receptor. sFLT-1 binds with very high affinity to VEGF without initiation of signal transduction and as such results in site-specific inhibition of the function of VEGF leading to regulation of angiogenic process in acute myeloid cells [17, 18].

 Gene therapy-based sFLT-1 in antiangiogenic strategies has been studied in various types of cancers using viral and nonviral carriers. However, few studies have elaborated the role of sFLT-1 in hematologic malignancies. In the current study, we investigated the effects of sFLT-1 gene delivery using a cationic lipid on acute myeloid leukemic cells. We constructed the sFLT-1 gene that codes the 1–3 immunoglobulin-(Ig-) like domains of FLT-1 and established a recombinant plasmid expressing sFLT-1. Up to our knowledge, there is no study related to the delivery of psFLT-1 in leukemic cells by mediating lipofectamine 2000. Therefore, for the first time, we investigated the delivery of pVAX-sFLT-1 in acute myeloid leukemic cells and explored the *in vitro* inhibitory effects of sFLT-1in K562 and HL60 cells as well as HUVECs. 

## 2. Materials and Methods

### 2.1. Cell Lines

HL-60 (human promyelocytic leukemic cells) and K562 (myelogenous leukemia, erythroleukemia type) were obtained from American Type Culture Collection (ATCC, Manassas, VA, USA) and were maintained in complete medium composed of RPMI supplemented with 10% fetal bovine serum (Hyclone Labratories, Austria). Human umbilical vein endothelial cells (HUVECs) were obtained from ATCC and maintained in F12K supplemented with endothelial cell growth supplement (ECGS) (0.05 mg/mL) (BD Biosciences, Bedford, MA, USA) and heparin (Sigma-Aldrich, Australia) (0.1 mg/mL). The media for cells were supplemented with 1% penicillin, streptomycin (Sigma-Aldrich, St Louis, MO, USA). All of the cells were grown at 37°C in a humidified atmosphere of 5% CO_2_. 

#### 2.1.1. Reverse Transcriptase-Polymerase Chain Reaction

Detection of VEGF165 and VEGFR-1 expression at the mRNA level in two leukemic cell lines was carried out prior to investigation of the effects of sFLT-1 delivery. RNA from cultured cells was isolated using MasterPure RNA Purification Kit according to the manufacturer's protocol (Epicentre, Madison, WI, USA). RNA (1 *μ*g) was processed for cDNA synthesis using RevertAid H Minus First Strand cDNA Synthesis Kit with random hexamer primer (Fermentas, Lithuania). The cDNA (100 ng) was used for polymerase chain reaction (PCR) in a final volume of 25 *μ*L using BIO-X-ACT Short Mix PCR reagent (Bioline, USA) in a thermal cycler gradient PCR system (Eppendorf, Germany). The primers were obtained from Sigma-aldrich, Singapore. The primers used for human VEGF-165: sense primer (5′-ACCCTGATGAGATCGA-GTAC-3′), antisense primer (5′-TAATCTGCATGGTGATG TTGG-3′), VEGFR-1: sense primer 5′-AC TCTA ACATGCACCTGTGTGG-3′, antisense primer 5′-GCTCATCCAAAGGAACT TCATCTG-3′, and Glyceraldehyde phosphate dehydrogenase (GAPDH); sense primer (5′-ACA ACAGCTCAAGATCATCAG-3′) antisense primer (5′-ATG AGT CCT TCC ACGA TACC-3′) 33 cycles, annealing at 55°C, amplified fragments of 567, 145, and 121 bp, respectively. PCR products were resolved on a 2% agarose gel and stained with ethidium bromide. The amplified DNA was visualized under UV light and images were obtained by the Alpha-Innotech gel documentation system (Stratagene, La Jolla, CA). 

### 2.2. Cloning of the 1–3 Ig-Like Domains of FLT-1

Total cellular RNA was isolated from HUVECs with MasterPure RNA Purification Kit (Epicenter Biotechnology, USA) according to the manufacturer's instructions. cDNA (Fermentas, Lithuania) was amplified using the primers designed to code to the 1–3 Ig-like domains of FLT-1 (accession no. X51602) [19]. The forward and reverse primers were modified to allow ligation into the pVAX1 plasmid at the *HindIII*–*ECORI* restriction sites as follows: forward 5′CC **AAG CTT** ACC ATG GTC AGC TA-3′ and reverse 5′-CG **GAA TTC** TTA ATA TGC ACT GAG-3′. The PCR reaction was run at 94°C for 4 min; 35 cycles at 94°C for 1 min; 67.4°C (VEGF165), 55°C (VEGFR-1, and GAPDH) for 50 s; and 72°C for 50 s following an extension for 10 min at 72°C. The resulting PCR product (1000 bp) was cloned into pVAX1 plasmid (Invitrogen, San Diego, USA), following digestion, ligation, and transformation of top10 *E. coli* cells, positive colonies were chosen by restriction digestion. Positive colonies were then subjected to DNA sequencing analysis (NHK Bio-Science, Korea) for final verification.

### 2.3. Transduction of K562 and HL60 Cells and Confirmation of sFLT-1 Expression

#### 2.3.1. Reverse Transcriptase-Polymerase Chain Reaction

The K562 and HL60 cells (1 × 10^6^cells/mL) in 25 cm^2^ tissue culture flasks (BD Biosciences Falcon) were transfected with pVAX-sFLT-1 and pVAX-GFP using lipofectamine 2000 (Invitrogen, San Diego, USA) according the manufacturer's instruction. After incubation at 37°C and 5% CO_2_ for 4 h, subsequently the transfection medium was replaced with complete growth media and incubated for 72 h. To confirm the expression of sFLT-1 by transfected cells, cellular RNA was isolated from the K562 and HL60 cells, cDNA was synthesized from total RNA, and then subjected to RT-PCR using the cloning primers described above. RT-PCR was performed using the BIO-X-ACT Short Mix PCR reagent (Bioline USA Inc, Taunton, MA, USA) with the same condition described earlier. 

### 2.4. Western Blot Analysis

Conditioned media from K562 and HL60 cells transfected with pVAX-sFLT-1 and pVAX-GFP 72 h posttransfection were subjected to western blot analysis. Concentrated conditioned media (6x) were electrophoretically separated on a 12% sodium dodecyl sulfate-polyacrylamide gel under reducing conditions. The gel was then transferred to polyvinylidene difluoride membranes (Millipore, Billerica, USA), probed by anti-VEGFR1 (FLT-1), Mouse IgG1 isotype (1/300) (R&D Systems Inc, Minneapolis, MN, USA), then reacted with alkaline phosphatase-conjugated secondary antibody Goat anti-mouse Ab 1/5000 (Chemicon, USA). The BCIP /NBT solution in alkaline phosphatase buffer (100 mM Tris, 100 mM NaCl, 5 mM MgCl_2_; pH 9.5) was added as a substrate and the bands were observed on the membrane. 

### 2.5. K562 and HL60 Cells Proliferation Inhibition Assay

The inhibitory effect of pVAX-sFLT-1 on K562 and HL60 cell proliferation was assessed using CellTiter 96 AQ_ ueous_ Non-Radioactive Cell Proliferation Assay (MTS) (Promega, Madison, USA), which was cleaved by viable cells to yield a dark blue formazan product. K562 and HL60 cells at a density of 1 × 10^4^ cells/well in 24-well plates were transfected with pVAX-sFLT-1 or pVAX-GFP using lipofectamine 2000. After 4 h, the infection media were replaced with serum-free medium (OptiMEM I Medium, Invitrogen Gibco, USA) and then 10 ng/mL recombinant human VEGF165 (R&D Systems, Minneapolis, USA) was added to each well and incubated for 72 h. The cells were transferred to 96-well plate and subjected to evaluate cell proliferation using MTS assay. The absorbance of formazan at 490 nm was determined on a multiwell plate reader.

### 2.6. HUVEC Proliferation Inhibition Assay

HUVECs were grown in 24-well plates at 37°C in complete growth media at 80% confluence; the concentrated conditioned media were obtained from K562 cells transfected with pVAX-sFLT-1 or pVAX-GFP in triplicates. HUVECs were washed with PBS, and 500 *μ*L conditioned medium 6x from transfected cells and untransfected cells was added to the related wells. After 15 min, recombinant human VEGF165 (R&D Systems, Minneapolis, USA) (10 ng/mL) was added to each well and incubated at 37°C. After 72 h, the cells were trypsinized and the proliferation of cells was estimated by MTS assay 96-well plates in absorbance of 490 nm. The experiments were performed in triplicate. Experiments were repeated three independent times.

### 2.7. HUVEC Migration Inhibition Assay

The inhibitory effects of sFLT-1 expression from K562 cells in the migration of HUVECs were investigated using QCM 24-well Colorimetric Cell Migration kit (Chemicon International, USA) utilizing an 8 *μ*m pore size. According to the instruction of the kit, the HUVECs were treated with conditioned media of K562 cells transfected with pVAX-sFLT-1 and pVAX-GFP for 48 h. Then, cells were harvested and transferred to inserts in triplicates and 500 *μ*L conditioned media (6x) obtained from transfected or untransfected cells, containing 40 ng/*μ*L recombinant human VEGF165 protein were added to the lower chamber and incubated for 24 h at 37°C in a CO_2_ incubator. After 24 h the migration inserts were subjected to staining and extraction steps according the instruction of kit. The extraction mixtures were transferred to a 96-well microtiter plate and the optical density measured at 560 nm. Experiments were repeated three independent times, each with triplicate reactions.

### 2.8. Statistical Analysis

All analyses were performed using SPSS 16.0. Before analysis, all data were subjected to Kolmogorov-Smirnove test for normality. To show the differences among groups, Anova was applied. Mean comparison was done using Duncan multiple range test (DMRT). The comparison of results between or among the groups was analyzed by paired sample *t*-test. Test results were considered statistically significant if the probability was less than 0.05.

## 3. Results

### 3.1. Expression of VEGF165 by Leukemic Cells

**Figure 1:**
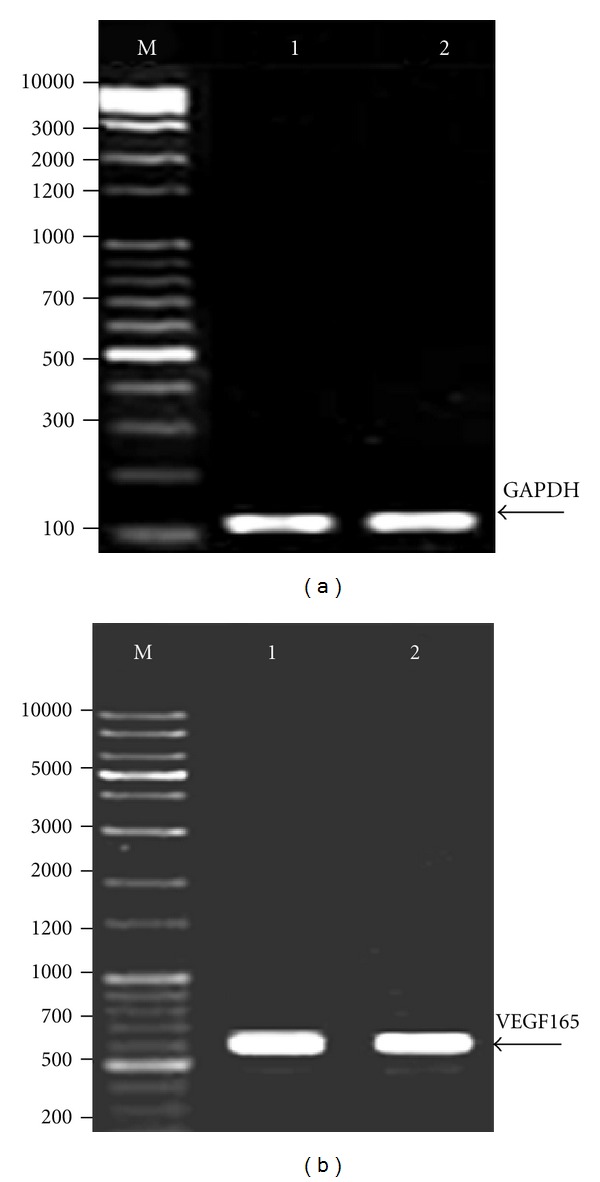
Identification of VEGF165 mRNA in K562 and HL60 cells using RT-PCR. (a) GAPDH served as an internal RNA loading control. Lane M: DNA marker (1 Kb plus 100 bp, Mbiotech, Korea); lanes 1 and 2: K562 and HL60 cells, respectively. Arrow indicates GAPDH at 121 bp, (b) lane M: DNA marker (1 Kb plus 100 bp, Mbiotech, Korea); lanes 1 and 2: K562 and HL60 cells, respectively. Arrow indicates VEGF165 at 567 bp.

**Figure 2:**
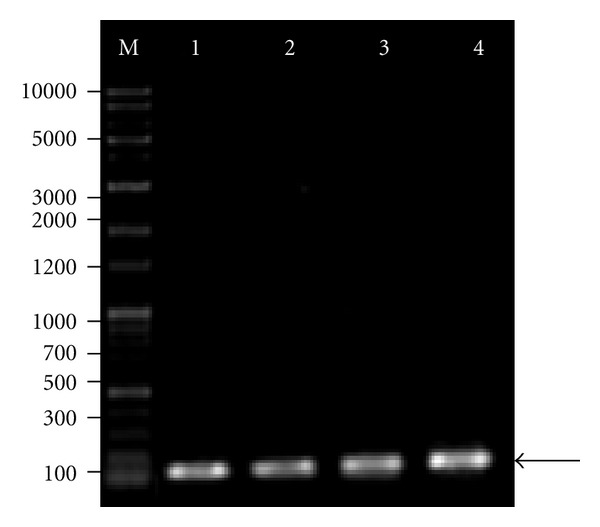
Identification of VEGFR-1 in K562 and HL60 cells using RT-PCR. GAPDH served as an internal RNA loading control. Lane M: DNA marker (1 Kb plus 100 bp, Mbiotech, Korea); lanes 1 and 2: K562 and HL60 cells, respectively; arrow indicates GAPDH PCR amplicon at 121 bp; lanes 3 and 4: K562 and HL60 cells, respectively; arrow indicates VEGFR-1 PCR amplicon at 145 bp.

Expression of VEGF165 and VEGFR-1 mRNA in leukemic cells was identified using RT-PCR analysis. The expression of GAPDH mRNA was evaluated to verify equal mRNA levels. The presence of two fragments, 567 bp (VEGF165) and 145 bp (VEGFR-1), was identified in agarose gel electrophoresis. (Figures 1 and 2). 

### 3.2. Construction of Recombinant pVAX-sFLT-1 Plasmid

**Figure 3:**
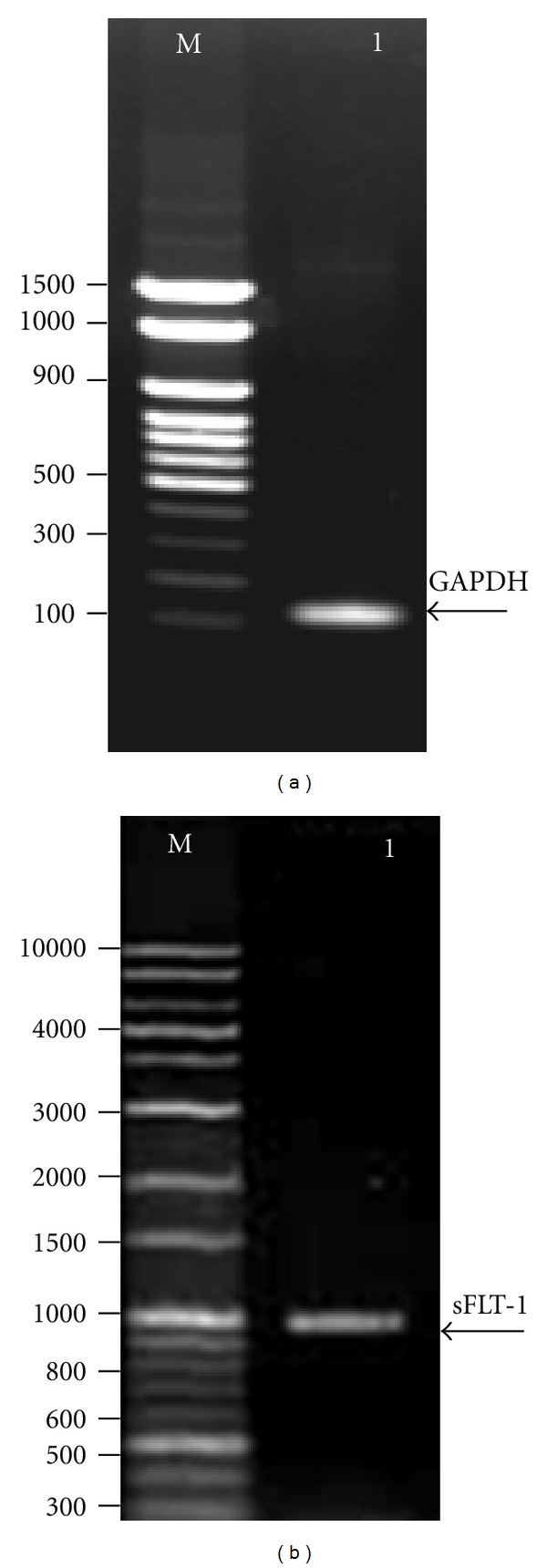
Identification of sFLT-1 from HUVEC. RT-PCR was performed using primers corresponding to human sFLT-1 and GAPDH cDNA sequences. GAPDH served as an internal RNA loading control. (a) Lane M: DNA marker (100 bp, Mbiotech, Korea); lane 1: arrow indicates GAPDH at 121 bp. (b) Lane M: DNA marker (1 Kb plus 100 bp, Mbiotech, Korea); lane 1: arrow indicates amplification of sFLT-1 at 1000 bp.

**Figure 4:**
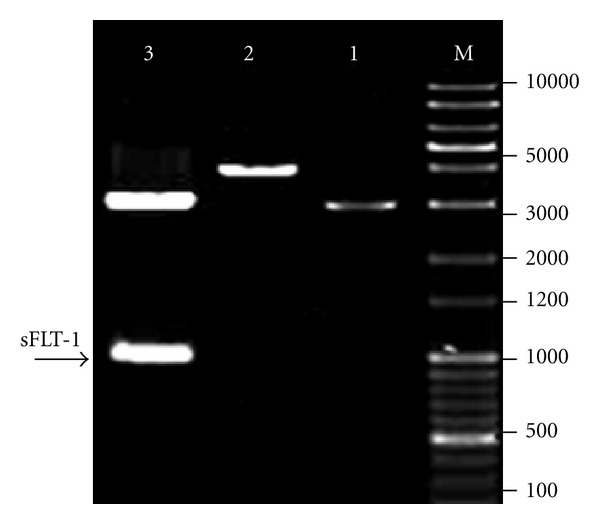
Identification of the recombinant pVAX1-sFLT-1 plasmid. Lane M: DNA marker (1 Kb plus 100 bp, Mbiotech, Korea); lane 1: digestion of plasmid pVAX1 (negative control) with *Hind III* (3000 bp); lane 2: digestion of recombinant plasmid with *Hind III* (4000 bp); lane 3: digestion of recombinant plasmid with *Hind III*+ *ECORI*, arrow indicates release of sFLT-1 fragment (1000 bp).

The sFLT-1 was amplified from HUVEC cDNA (Figure 3) and was cloned into a pVAX plasmid. Then, recombinant plasmid expressing sFLT-1 (pVAX-sFLT-1) was generated and endonuclease digestion of recombination plasmids (Figure 4) confirmed the presence of sFLT-1. 

### 3.3. Expression of sFLT-1

**Figure 5:**
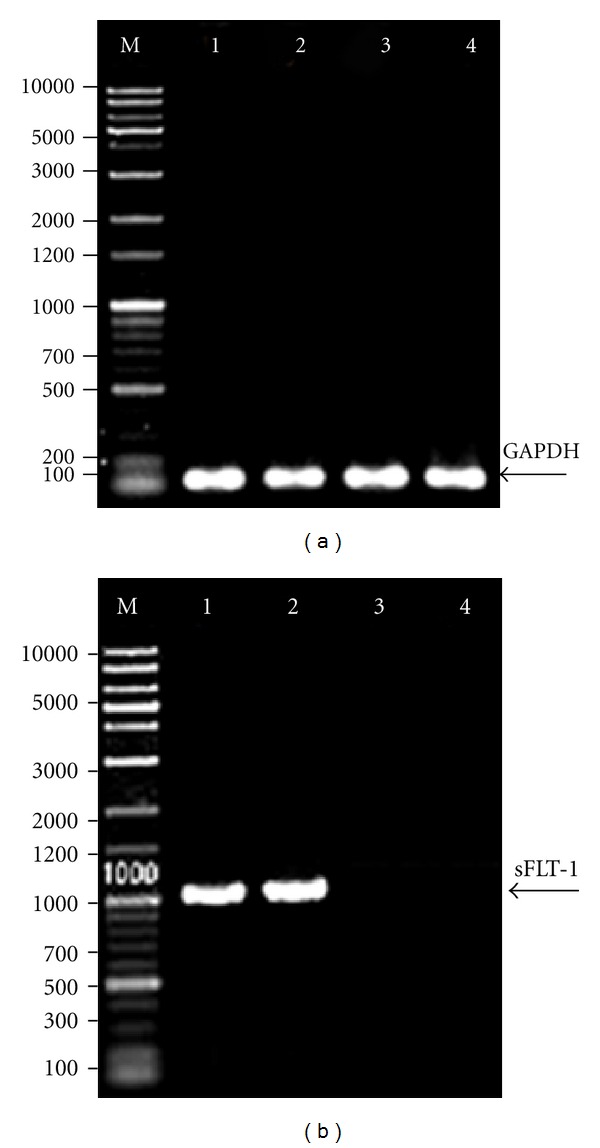
Identification of sFLT-1 mRNA from K562 and HL60 cells using RT-PCR. (a) GAPDH served as an internal RNA loading control. Lane M: DNA marker (1 Kb plus 100 bp, Mbiotech, Korea); lanes 1 and 2: K562 and HL60 cells transfected with pVAX1-sFLT-1, respectively; lanes 3 and 4: K562 and HL60 cells transfected with pVAX1-hMGFP. (b) Lane M: DNA marker (1 Kb plus 100 bp, Mbiotech, Korea); lanes 1 and 2: K562 and HL60 cells transfected with pVAX1-sFLT-1; lanes 3 and 4: K562 and HL60 cells transfected with pVAX1-hMGFP. Arrows indicate expression of GAPDH at 121 bp and sFLT-1 at 1000 bp.

**Figure 6:**
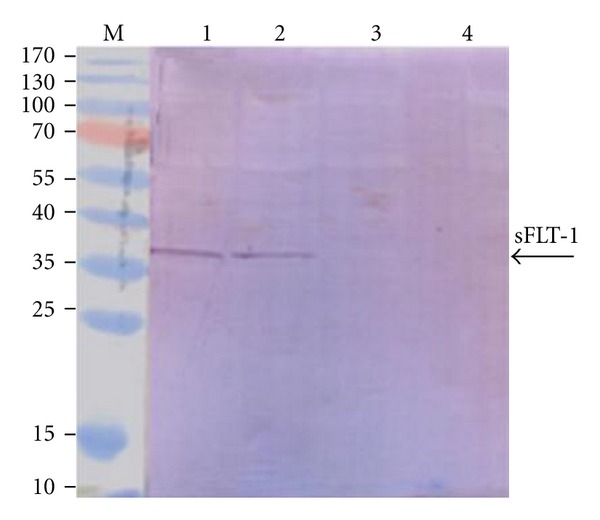
Secretion of sFLT-1 in conditioned media of pVAX1-sFLT-1 transfected cells. (a) Western blot analysis with anti-mouse VEGFR-1 antibody, similar results were observed as arrow indicates. Lane M: (PageRuler Prestained Protein Ladder, Fermentas, Lithuania); lanes 1 and 2: K562 and HL60 cells transfected with pVAX1-sFLT-1; lanes 3 and 4: K562 and HL60 cells transfected with pVAX1-hMGFP (negative control).

Transfection of K562 and HL60 cells with pVAX-sFLT-1 and pVAX-GFP by lipofectamine resulted in the expression/secretion of sFLT-1. The cell lysate and conditioned media of transfected cells were subjected to RT-PCR and western blot analysis, respectively. As the Figure 5 shown, sFLT-1mRNA at size of 1000 bp was amplified from cDNA of pVAX-sFLT-1 transfected cell but not pVAX-GFP. The western blot analysis demonstrated that sFLT-1 was expressed and secret in conditioned media from pVAX-sFLT-1 at size of 37 KDa (Figure 6). The transgene expression by recombinant plasmid was evaluated in time points 48 and 72 h posttransfection using a recombinant plasmid expressing GFP, but the results were not shown.

### 3.4. *In Vitro* Inhibitory Effect of sFLT-1 in Proliferation of K562 and HL60 Cells

**Figure 7:**
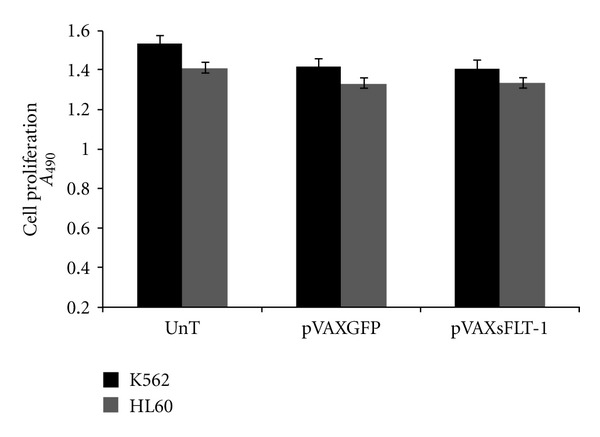
*In vitro* inhibition of K562 and HL60 proliferation by sFLT-1. K562 and HL60 cells transfected with psFLT-1 subjected to MTS analysis. psFLT-1 transfected cells versus pGFP transfected or untreated cells. Each data point is presented as mean ± SEM. The *P* value greater than 0.05 (0.349 and 0.769, resp., in K562 and HL60 treated cells) showed no significant inhibitory effect in treated cells compared with control groups.

Inhibitory effect of sFLT-1 expressing from pVAX-sFLT-1 transfected K562 and HL60 cells was evaluated using MTS proliferation analysis. As shown in Figure 7, sFLT-1 had no inhibitory effect on leukemic cells growth in the presence of VEGF after 72 h as the results were compared with pVAX-GFP transfected or untransfected cells (*P* > 0.05). 

### 3.5. *In Vitro* Inhibitory Effect of sFLT-1 in Proliferation of HUVEC

**Figure 8:**
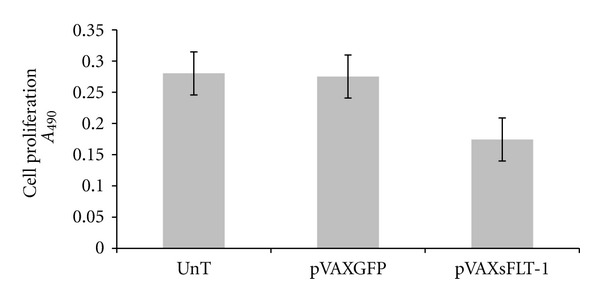
*In vitro* inhibition of HUVEC proliferation by sFLT-1. sFLT-1 treated HUVEC with conditioned media of K562-sFLT-1 was subjected to MTS analysis. sFLT-1 treated cells versus GFP treated or untreated cells. Each data point is presented as mean ± SEM. Test results were considered statistically significant as *P* value (0.001) was less than 0.05. Results indicate the rate of cell proliferation in HUVEC affected by sFLT-1.

The functional confirmation of secreted sFLT-1 mediating pVAX-sFLT-1 was evaluated by HUVEC inhibition analysis using MTS assay. As shown in Figure 8, incubation of HUVECs with conditioned media K562 cells expressing sFLT-1 after 72 h inhibited proliferation of HUVECs by 40% when compared with HUVECs incubated with conditioned media from pVAX-GFP and untransfected cells, respectively.

### 3.6. *In Vitro* Inhibitory Effect of sFLT-1 on Migration of HUVEC

**Figure 9:**
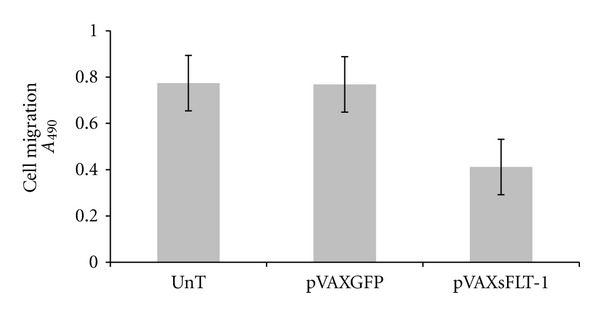
*In vitro* inhibition of HUVEC migration by sFLT-1. The optical density of migrated cells was measured at 490 nm. Results indicate migration of sFLT-1 treated cells versus GFP treated or untreated cells. Each data point is presented as mean ± SEM. Considerable decrease in cell migration was observed as *P* value (0.001) was less than the chosen significance level (0.05).

HUVECs migration was evaluated after treatment of cells with conditioned media of pVAX-sFLT-1 transfected. HUVECs were incubated with conditioned media from pVAX-sFLT-1, pVAX-GFP, and untransfected cells and applied to migration assay using QCM 24-well Colorimetric Cell Migration kit in presence of VEGF (40 ng/mL). As shown in Figure 9, conditioned media from sFLT-1 expressing cells inhibited migration of HUVECs by 43% compared with conditioned media from pVAX-GFP and untransfected cells, respectively.

## 4. Discussion

Several approaches were investigated for the effectiveness in delivering sFLT-1 gene therapy, including naked plasmid DNA [20], HVJ-cationic liposome-formulated plasmid DNA, and adenoviral-mediated approaches [21]. Most of these studies have evaluated delivery of sFLT-1 in solid tumours, including a peritoneal metastasis model using human fibro-sarcoma cells [21], ovarian cancer [22], follicular thyroid carcinoma [19], and multiple myeloma [23]. These studies indicated that sFLT-1 expression caused a significant inhibition in endothelial cell proliferation *in vitro* and the suppression of tumour growth and metastasis, as well as longer survival period. Also, it is determined that the systemic delivery of the sFLT-1 mediated by mammalian cells could be effective to inhibit tumour growth and angiogenesis [19]. These studies were focused on solid tumours. Only one study was performed on haematologic malignancies, which is multiple myeloma (MM), demonstrating that the systemic delivery of ADV-sFLT effectively inhibited MM growth through decreasing the microvessel density of tumours. It is worth mentioning that the authors of that study found that the biological effect of sFLT-1 in multiple myeloma was mainly through inhibiting the angiogenesis of the tumour without affecting its growth. These findings present the effect of sFLT-1 on VEGF-mediated paracrine loop [23]. Most of studies have delivered sFLT-1 using viral vectors; however, due to immune response concerns related to viral vectors, using nonviral gene carriers, which are not immunogenic, can be effective approach for delivery of such inhibitors. Therefore, we have developed an approach to inhibit angiogenesis through inhibition of VEGF with its natural inhibitor, sFLT-1. To this end, one of the most widely used cationic gene carriers, Lipofectamine 2000, was used for delivering therapeutic gene into angiogenic endothelial cells. The human sFLT-1 molecule constructed in the current study contained the extracellular domains 1–3 of the FLT-1 receptor and was able to effectively block angiogenesis and tumor growth. Barleon et al. have identified that there is no difference between the affinity of full-length extracellular domains and the 1–3 extracellular domains of sFLT-1 mutants in binding VEGF, and the first three Ig-like loops are able to compete efficiently with VEGF165 [24].

To our knowledge, to date, there is no study related to delivery of psFLT-1 in leukemic cells mediating lipofectamine 2000. Here, we investigated inhibitory effect of sFLT-1 myeloid leukemic cells and human umbilical endothelial cells. The results demonstrated that although sFLT-1 did not inhibit cell proliferation of the leukemic cells, it could significantly suppress the proliferation and migration of HUVEC after incubation with conditioned media K562 cells expressing sFLT-1. Interestingly, the same results were observed in the study by Liu et al. They determined that the proliferation of human multiple myeloma cells (KM3) transduced with ADV-sFLT was not inhibited in the presence or absence of VEGF, while the conditioned media of transduced cells could significantly inhibit the growth of HUVECs. Moreover, they found that ADV-sFLT-1 exerted its inhibitory effect on human MM tumours mainly through inhibiting the angiogenesis of the tumour and decreasing the microvessel density of tumours, without affecting the tumour cell proliferation directly [23]. Also, Kim et al. showed that PEI-g-PEG-1.3RGD/pCMV-sFLT-1 delivery in colon adenocarcinoma cells had no inhibitory effect on the growth of colon adenocarcinoma cells, whereas the transfection of primary endothelial cells (CADMEC) with PEI-g-PEG-1.3RGD/pCMV-sFLT-1 resulted in the suppression of VEGF-driven proliferation of endothelial cells. Therefore, they exhibited significant selectivity and effectiveness of PEI-g-PEG-RGD/pCMV-sFLT-1 complexes in an endothelial cell inhibition assay by abrogating VEGF effects through the binding sFLT-1 [25]. On the other hand, other studies which focused on the sFLT-1 gene delivery in solid tumours reported only the inhibitory effect of sFLT-1 on the proliferation of endothelial cells *in vitro*, while the inhibitory effect of sFLT-1 on the VEGF-mediated proliferation of tumour cells *in vitro* was not studied [19, 22]. 

Studies showed that expression of VEGEF and its receptors on myeloid leukemic cells contributed to the growth and survival of leukemic blasts through the autocrine loop [7]. Also, the role of the VEGF-mediated loop in the progression of myeloid leukemic cells was investigated by the application of an antisense-VEGF in K562 cells. The silencing of VEGF by an antisense-VEGF in K562 cells inhibited proliferation of K562 cell through increasing cell apoptosis [26]. To the contrary, in the current study, although the expression of VEGF and VEGFR-1 in K562 and HL60 cells was identified, inhibition of the VEGF-mediated autocrine loop was not observed by sFLT-1 in these cells. Consequently, these results are in agreement with previous studies that propose VEGF-mediated inhibitory effect of sFLT-1 trough paracrine loop. This means that sFLT-1 is probably involved in the survival and progression of leukemic blasts by its inhibitory effects on marrow endothelial cells (paracrine loop). This statement was concluded in the study by Liu et al. on multiple myeloma. To explain this behaviour, it is mentioned that the solubility of sFLT-1 probably allows it to act in a paracrine manner on vascular endothelial cell receptors [23]. Taken together, more investigations may be needed to explain and understand the effect of sFLT-1 gene delivery on VEGF-derived proliferation and the survival of leukemic blasts. 

## 5. Conclusion

In agreement with other studies, no link between the proliferation of K562 and HL60 cells and the inhibitory effect of sFLT-1was detected. However, it was found that sFLT-1 expression from K562 cells can inhibit significantly the VEGF-driven proliferation and migration of HUVECs *in vitro*. We suggest that although sFLT-1 could not affect the proliferation of K562 and HL60 cells in a VEGF-mediated autocrine loop, it may play an inhibitory role by affecting VEGF-mediating paracrine loop.

## References

[1] Griffin JD, Lowenberg B (1986). Clonogenic cells in acute myeloblastic leukemia. *Blood*.

[2] Estey E, Döhner H (2006). Acute myeloid leukaemia. *The Lancet*.

[3] Hanahan D, Folkman J (1996). Patterns and emerging mechanisms of the angiogenic switch during tumorigenesis. *Cell*.

[4] Folkman J (1995). Clinical applications of research on angiogenesis. *The New England Journal of Medicine*.

[5] Risau W (1997). Mechanisms of angiogenesis. *Nature*.

[6] Hussong JW, Rodgers GM, Shami PJ (2000). Evidence of increased angiogenesis in patients with acute myeloid leukemia. *Blood*.

[7] Ferrara N (1999). Role of vascular endothelial growth factor in the regulation of angiogenesis. *Kidney International*.

[8] Frankel AE, Gill PS (2004). VEGF and myeloid leukemias. *Leukemia Research*.

[9] Barbarroja N, Arístides-Torres L, Hernandez V (2007). Coordinated deregulation of cellular receptors, proangiogenic factors and intracellular pathways in acute myeloid leukaemia. *Leukemia and Lymphoma*.

[10] Aguayo A, Estey E, Kantarjian H (1999). Cellular vascular endothelial growth factor is a predictor of outcome in patients with acute myeloid leukemia. *Blood*.

[11] Schuch G, Machluf M, Bartsch G (2002). In vivo administration of vascular endothelial growth factor (VEGF) and its antagonist, soluble neuropilin-1, predicts a role of VEGF in the progression of acute myeloid leukemia in vivo. *Blood*.

[12] Padró T, Ruiz S, Bieker R (2000). Increased angiogenesis in the bone marrow of patients with acute myeloid leukemia. *Blood*.

[13] Wegiel B, Ekberg J, Talasila KM, Jalili S, Persson JL (2009). The role of VEGF and a functional link between VEGF and p27Kip1 in acute myeloid leukemia. *Leukemia*.

[14] Grimwade D (2001). The clinical significance of cytogenetic abnormalities in acute myeloid leukaemia. *Best Practice and Research*.

[15] Kelly LM, Gilliland DG (2002). Genetics of myeloid leukemias. *Annual Review of Genomics and Human Genetics*.

[16] Kume A, Hanazono Y, Mizukami H, Okada T, Ozawa K (2002). Selective expansion of transduced cells for hematopoietic stem cell gene therapy. *International Journal of Hematology*.

[17] Toi M, Bando H, Ogawa T, Muta M, Hornig C, Weich HA (2002). Significance of vascular endothelial growth factor (VEGF)/soluble VEGF receptor-1 relationship in breast cancer. *International Journal of Cancer*.

[18] Lamszus K, Ulbricht U, Matschke J, Brockmann MA, Fillbrandt R, Westphal M (2003). Levels of soluble vascular endothelial growth factor (VEGF) receptor 1 in astrocytic tumors and its relation to malignancy, vascularity, and VEGF-A. *Clinical Cancer Research*.

[19] Ye C, Feng C, Wang S (2004). sFlt-1 gene therapy of follicular thyroid carcinoma. *Endocrinology*.

[20] Goldman CK, Kendall RL, Cabrera G (1998). Paracrine expression of a native soluble vascular endothelial growth factor receptor inhibits tumor growth, metastasis, and mortality rate. *Proceedings of the National Academy of Sciences of the United States of America*.

[21] Kong HL, Hecht D, Song W (1998). Regional suppression of tumor growth by in vivo transfer of a cDNA encoding a secreted form of the extracellular domain of the flt-1 vascular endothelial growth factor receptor. *Human Gene Therapy*.

[22] Mahasreshti PJ, Navarro JG, Kataram M (2001). Adenovirus-mediated soluble FLT-1 gene therapy for ovarian carcinoma. *Clinical Cancer Research*.

[23] Liu J, Li J, Su C, Huang B, Luo S (2007). Soluble fms-like tyrosine kinase-1 expression inhibits the growth of multiple myeloma in nude mice. *Acta Biochimica et Biophysica Sinica*.

[24] Barleon B, Totzke F, Herzog C (1997). Mapping of the sites for ligand binding and receptor dimerization at the extracellular domain of the vascular endothelial growth factor receptor FLT- 1. *Journal of Biological Chemistry*.

[25] Kim WJ, Yockman JW, Lee M, Jeong JH, Kim YH, Kim SW (2005). Soluble Flt-1 gene delivery using PEI-g-PEG-RGD conjugate for anti-angiogenesis. *Journal of Controlled Release*.

[26] He R, Liu B, Yang C, Yang RC, Tobelem G, Han ZC (2003). Inhibition of K562 leukemia angiogenesis and growth by expression of antisense vascular endothelial growth factor (VEGF) sequence. *Cancer Gene Therapy*.

